# Doxorubicin-Loaded Nanoparticle Treatment Enhances Diffuse Large B-Cell Lymphoma Cell Death

**DOI:** 10.3390/cells14171334

**Published:** 2025-08-28

**Authors:** Ihab Abd-Elrahman, Noha Khairi, Taher Nassar, Riki Perlman, Dina Ben Yehuda

**Affiliations:** 1 Department of Hematology, Hadassah Medical Center, Jerusalem 9112001, Israel; ihabae@savion.huji.ac.il (I.A.-E.); noharabieh89@gmail.com (N.K.); priki@hadassah.org.il (R.P.); 2 Faculty of Medicine, The Hebrew University of Jerusalem, Jerusalem 9112001, Israel; tahern@ekmd.huji.ac.il

**Keywords:** drug delivery, PLGA nanoparticles, oleyl cysteineamide, doxorubicin, DLBCL, cancer therapy

## Abstract

Drug resistance remains a major obstacle in cancer treatment despite advances in therapeutic regimens. To address this, we explored the potential of Doxorubicin (Dox) delivery in poly (lactide-co-glycolic acid) (PLGA) nanoparticles to enhance Diffuse large B-cell lymphoma (DLBCL) cell death. This research investigates the potential of Doxorubicin and advanced delivery methods. We used PLGA nanoparticles with Oleyl cysteineamide (OCA); its amphiphilic nature enables interfacial anchoring and thiol surface functionalization of PLGA NPs. Compared to PLGA-NPs, PLGA-OCA-NPs enhance immunity and induce tumor cell death. They also show significant apoptotic cell death and induced immune responses in DLBCL mouse models. Dox-conjugated PLGA-OCA-NPs (DOX-OCA) exhibit significant in vitro and in vivo anticancer activity compared to free DOX, showing remarkable antitumor effects with reduced systemic toxicity in mouse models. Our findings underscore the promising potential of PLGA-OCA-NPs in DLBCL treatment, offering a hopeful future in cancer therapy. This innovative delivery system offers enhanced immune responses and effectively addresses toxicity concerns, marking a significant step forward in cancer therapy.

## 1. Introduction

Significant progress has been made in developing new cancer treatment regimens, but the emergence of drug resistance remains a major obstacle to achieving durable remissions in many malignancies [1]. Extensive efforts have been devoted to understanding the mechanisms behind the eventual failure of initially promising targeted therapies [2].

Diffuse large B-cell lymphoma (DLBCL) is the most common aggressive lymphoma, accounting for roughly 30–40% of non-Hodgkin lymphoma cases at diagnosis [3].

While first-line chemoimmunotherapy (e.g., R-CHOP) can induce long-term remissions, a substantial subset of patients, about 30% of DLBCL patients, either fail to respond or relapse with treatment-refractory disease [4]. New therapeutic approaches are therefore urgently needed, especially for high-risk patients.

Several novel immunotherapeutic strategies, such as bispecific T-cell engager (BiTE^®^) antibodies and chimeric antigen receptor (CAR) T-cell therapies, have shown promise for DLBCL patients who do not respond to R-CHOP. However, even with these advances, approximately 50% of refractory DLBCL cases still do not achieve adequate remission [5]. Thus, there remains a pressing need for alternative targeted treatments for patients who are unresponsive to conventional chemotherapy.

Doxorubicin (DOX) is a key component of first-line therapy for DLBCL and many other cancers. Its antitumor activity is multifaceted; DOX intercalates into DNA and disrupts transcription, generates reactive oxygen species, and inhibits topoisomerase II, all of which contribute to cancer cell death [6]. In addition, DOX can activate specific cellular pathways; for example, it triggers the release of the membrane-bound transcription factor CREB3L1, and tumor cells with high CREB3L1 expression are especially sensitive to DOX treatment [7,8].

Interestingly, the mode of DOX administration can influence the cellular response. A bolus high-dose exposure to DOX induces G2 cell-cycle arrest with p53 phosphorylation and upregulation of pro-apoptotic factors (such as Bax and p21) leading to pronounced apoptosis, whereas continuous low-dose exposure results in significantly less apoptosis [9].

Despite its efficacy, the clinical utility of DOX is limited by dose-dependent cardiotoxicity. The incidence of DOX-induced congestive heart failure increases from around 4% at cumulative doses of 500–550 mg/m^2^ to about 18% at 551–600 mg/m^2^ and rises to ~36% when the total dose exceeds 600 mg/m^2^ [10]. Consequently, various strategies have been explored to mitigate DOX cardiotoxicity, including the use of liposomal DOX (Doxil) formulations that alter drug pharmacokinetics and distribution [11,12].

Nanoparticle (NP)-based drug delivery systems have emerged as a promising strategy to improve the therapeutic index of chemotherapeutics by protecting drugs from premature degradation, enhancing tumor-specific biodistribution, providing controlled release, and reducing systemic toxicity. Among the many polymers evaluated for NP formulation, poly (lactic-co-glycolic acid) (PLGA) stands out due to its excellent safety profile, biodegradability, and FDA approval for clinical use. PLGA NPs gradually hydrolyze into lactic and glycolic acid monomers, which are naturally metabolized and eliminated via normal physiological pathways, minimizing the risk of long-term polymer accumulation and toxicity [13]. Furthermore, PLGA NPs can be engineered at sub-200 nm size, enabling passive tumor targeting via the enhanced permeability and retention (EPR) effect; these nanoscale particles preferentially extravasate into tumor tissue through leaky tumor vasculature [14,15]. In addition, delivering DOX within PLGA NPs may harness the drug’s immunomodulatory effects. DOX has been reported to stimulate tumor antigen presentation by dendritic cells, activate cytotoxic and helper T lymphocytes, and reduce immunosuppressive myeloid-derived suppressor cells (MDSCs) in the tumor microenvironment [16,17]. Therefore, PLGA represents a strategically optimal choice for NP-based chemotherapy formulations, balancing safety, efficacy, and potential immunological benefits.

Given the role of the immune system in eliminating cancer cells and inducing potent antitumor responses, a combination of chemotherapy and immunotherapy was explored in this study. Specifically, we investigated the therapeutic potential of PLGA nanoparticles, functionalized with OCA, for the delivery of DOX. These PLGA-OCA nanoparticles possess amphiphilic properties that facilitate interfacial anchoring and thiol surface functionalization, enhancing their bioactivity. The study focused on developing and characterizing DOX-loaded PLGA-OCA nanoparticles (DOX-OCA), evaluating their apoptotic and cytotoxic effects in DLBCL cell lines, and assessing their antitumor efficacy and systemic toxicity in a DLBCL xenograft model compared to free DOX and unmodified PLGA-NPs. In addition, the immunomodulatory capacity of PLGA-OCA-NPs was examined through the measurement of interferon-γ levels and peripheral immune cell profiling. This approach aims to improve the therapeutic index of DOX by enhancing tumor-targeted delivery, reducing off-target toxicity, and promoting immune-mediated tumor suppression.

## 2. Materials and Methods

### 2.1. Materials

Cell culture reagents. Iscove’s Modified Dulbecco’s Medium (IMDM; Gibco, Thermo Fisher Scientific, Waltham, MA, USA); fetal calf serum (FCS, 10%; Gibco); l-glutamine (1 mmol L^−1^; Gibco); penicillin (100 U mL^−1^; Gibco); streptomycin (100 µg mL^−1^; Gibco). Apoptosis/cell-death reagents. Annexin V-CY5 (Abcam, Cambridge, UK); propidium iodide (PI; Sigma-Aldrich, St. Louis, MO, USA). Caspase assay. CaspACE™ colorimetric caspase-3 assay kit (Promega, Madison, WI, USA). Nanoparticle formulation reagents. Poly(lactide-co-glycolide) (PLGA, Resomer RG 504H; MW ≈ 50 kDa; lactide:glycolide 50:50; Merck, Darmstadt, Germany); Oleyl cysteineamide (OCA; synthesized in-house; Solutol^®^ HS 15 (BASF, Ludwigshafen, Germany); doxorubicin hydrochloride (Sigma-Aldrich); acetone (HPLC grade; Sigma-Aldrich); trimethylamine (Sigma-Aldrich); Vivaspin™ (Merck, Darmstadt, Germany) centrifugal filters, 300 kDa MWCO (Sartorius, Göttingen, Germany). Animal study reagents and equipment. Male NOD.CB17-Prkdc^scid/J (NOD/SCID) mice, 7–8 weeks, Charles River Laboratories (Wilmington, MA, USA); IFN-γ sandwich ELISA kit (PeproTech, Rocky Hill, NJ, USA); BC-2800 auto-hematology analyzer (Mindray, Shenzhen, China); Tecan Infinite M200 Pro plate reader (Tecan, Männedorf, Switzerland).

### 2.2. Cells

OCI-Ly19 cells (a human DLBCL cell line, kindly provided by Dr. Neta Goldschmidt) were maintained in IMDM supplemented with 10% FCS, 100 U/mL penicillin, 100 µg/mL streptomycin, and 1 mM *L*-glutamine. All cell cultures were grown at 37 °C in a humidified atmosphere of 5% CO_2_.

### 2.3. Apoptosis and Cell Death Analysis

Apoptotic cell death was assessed by Annexin V/PI staining. Briefly, 5 × 10^5^ cells were incubated with the indicated treatments (nanoparticles or free drug conditions) for 48 h. The cells were then harvested, washed with PBS, and resuspended in an annexin V binding buffer. Cells were stained with Annexin V–Cy5 (1 µg/mL) for 15 min in the dark, washed, and counterstained with PI (0.5 µg/mL) just before analysis. Stained cells were immediately analyzed by flow cytometry (BD FACSCalibur) to quantify the percentages of apoptotic (Annexin V^+^/PI^−^) and dead (Annexin V^+^/PI^+^) cells.

### 2.4. Caspase-3 Activity Assay

Subcutaneous tumor tissues were collected from mice and homogenized as described previously [18]. Caspase-3 activity in each sample was then measured using a colorimetric caspase-3 assay kit (CaspACE™, Promega). In brief, 50 µg of total protein from each tumor lysate was incubated with the caspase-3 substrate according to the manufacturer’s instructions, and the resulting color change (reflecting enzymatic activity) was quantified by absorbance reading, following the kit protocol.

### 2.5. Preparation of PLGA Nanoparticles (NPs)

PLGA nanoparticles were prepared by a well-established interfacial deposition (nanoprecipitation) method [19]. In brief, 150 mg of PLGA (MW ~50 kDa) and 5 mg of OCA (Oleyl cysteineamide, synthesized in-house per Karra et al. 2013 [20]) were dissolved in 25 mL of acetone to form the organic phase. This solution was added dropwise under stirring to 50 mL of an aqueous phase containing 50 mg of Solutol^®^ HS 15 surfactant, with constant stirring at 900 rpm for 30 min. Nanoparticles formed upon the solvent displacement; thereafter, the organic solvent (acetone) was removed by rotary evaporation. The resultant NP suspension was adjusted to pH 6.5–7.0 and stored at 4 °C until use.

### 2.6. Preparation of Doxorubicin-Loaded NPs

To incorporate DOX into the formulation, 200 µL of trimethylamine and 100 mg of DOX-HCl were added to the PLGA-OCA organic phase prior to nanoparticle formation. The mixture was stirred at 900 rpm for 30 min, then processed as above to evaporate the solvent and allow nanoparticle assembly, yielding DOX-loaded PLGA-OCA nanoparticles (DOX-OCA). Unencapsulated (free) DOX was removed from the suspension by ultrafiltration using 300 kDa cutoff centrifugal filters (Vivaspin™) at 4500 rpm, with three successive wash cycles in PBS. The efficiency of DOX encapsulation was determined by measuring the absorbance of the filtrate and retentate at 475 nm (the absorbance maximum of DOX) and calculating the amount of drug incorporated into the NPs.

### 2.7. In Vivo DLBCL Subcutaneous Xenograft Model

All animal experiments were approved by the Institutional Animal Care and Use Committee (Protocol No. MD-16-14998-5) and were conducted in accordance with institutional ethical guidelines. Male NOD/SCID mice (7–8 weeks old) were housed under specific pathogen-free conditions with food and water provided ad libitum. To establish the DLBCL xenograft model, each mouse was injected subcutaneously into the right flank with OCI-Ly19 human DLBCL cells (5 × 10^6^ cells in PBS). This NOD/SCID–OCI-Ly19 model recapitulates an aggressive, treatment-resistant lymphoma and retains innate immune effectors (natural killer cells), thus providing a clinically relevant platform to evaluate enhanced DOX delivery strategies while also observing immune-related effects. Tumor growth was monitored every 2–3 days using digital calipers, and tumor volume was calculated as (length × width^2^)/2. Mice were also weighed regularly to assess systemic toxicity. Animals were humanely euthanized if a tumor exceeded 1.5 cm in diameter or if the mice showed signs of distress. Once tumors reached approximately 0.3 cm in diameter (around day 4 post-injection), mice were randomly assigned to treatment groups (n = 4–6 per group) using a blinded allocation method to ensure unbiased group distribution. To assess the efficacy and toxicity of free DOX, several groups of tumor-bearing mice were given free DOX intravenously at doses of 1.25, 2.5, or 5 mg/kg. Mice in the 1.25 mg/kg and 2.5 mg/kg treatment groups received DOX once weekly for three weeks, on days 4, 11, and 20 after tumor inoculation. Mice treated with 5 mg/kg received the drug on days 4 and 11 post-tumor inoculation. To assess the nanoparticle formulations, other groups of mice were treated intravenously on the same schedule (days 4, 11, 20) with one of the following: (i) unmodified PLGA-NPs (containing 0.5 mg of PLGA per injection), (ii) OCA-functionalized PLGA NPs without drug (PLGA-OCA NPs, 0.5 mg per injection), or (iii) DOX-conjugated PLGA-OCA NPs (DOX-OCA, delivering 1.25 mg/kg of DOX attached to 0.5 mg of PLGA-OCA per dose). All treatments were administered via the tail vein. One day after the third treatment (day 21 of the experiment), approximately 200 µL of blood was collected from each mouse via the facial vein (into EDTA-coated tubes). A complete blood count was then performed using an automated hematology analyzer (Mindray BC-2800) to determine the percentages of neutrophils, lymphocytes, and monocytes in peripheral blood as indicators of systemic immunological effects of the treatments.

### 2.8. Immune Activation Assay

To assess systemic cytokine responses, additional blood samples were collected at designated time points and processed to obtain serum. Serum interferon-γ (IFN-γ) levels were measured using a quantitative sandwich ELISA kit (PeproTech) according to the manufacturer’s instructions. In each assay, 5 µL of serum were used for IFN-γ detection. The colorimetric reaction was developed and measured by reading absorbance at 450 nm on a Tecan Infinite M200 Pro plate reader. IFN-γ concentrations were then calculated from a standard curve provided by the kit.

### 2.9. Statistical Analysis

Data are presented as mean ± standard error (SE). Statistical comparisons between groups were made using two-tailed Student’s *t*-tests. A *p*-value less than 0.05 was considered statistically significant.

## 3. Results

### 3.1. In Vivo Anticancer Activity and Cytotoxic Effect of Dox

The OCI-Ly19 cell line employed in this study is a well-established, clinically relevant model of diffuse large B-cell lymphoma (DLBCL). Genomic profiling classifies OCI-Ly19 as a germinal-center B-cell-like (GCB) subtype—one of the two principal DLBCL molecular classes—representing a large fraction of patient cases [21,22]. Critically, OCI-Ly19 carries concurrent MYC and BCL2 rearrangements (“double-hit”), recapitulating an aggressive, high-risk phenotype associated with poor prognosis [23]. The line retains a wild-type TP53 background, which is pertinent for evaluating DOX because p53 integrity influences DOX-induced apoptosis. Owing to these features and its extensive use in lymphoma research [21,22,23], OCI-Ly19 offers a rigorous and reproducible platform for testing novel DOX-loaded PLGA nanoparticles in a biologically defined, high-risk DLBCL context. Although the current work focuses on this single cell line, its molecular characteristics provide a meaningful first step before expanding validation to additional DLBCL subtypes.

**Figure 1:**
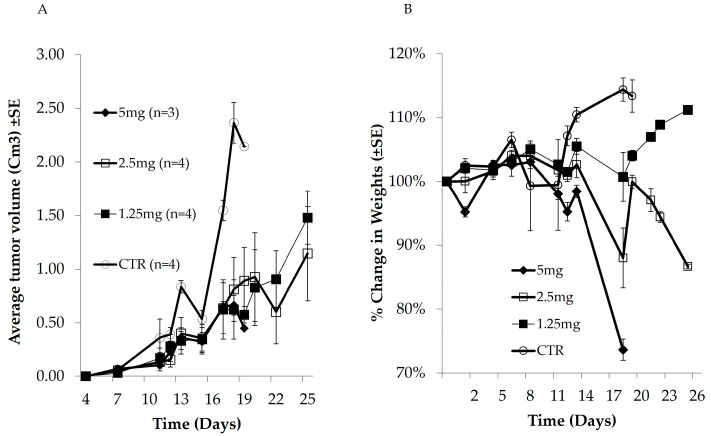
DLBCL tumors were induced in mice as described above. Mice were treated with different doses of DOX on days 4, 11, and 20 post-xenografts. Tumor volume (**A**) and body weight (**B**) were measured at the indicated time points. All mice in the control and 5 mg DOX groups were sacrificed on day 19.

Mice were injected subcutaneously with OCI-Ly19-DLBCL cells, and tumor volume was measured. In this model, tumors develop 7 days after injection of cells [24]. Three groups of mice were treated with different doses of DOX (1.25, 2.5, or 5 mg/kg). Mice treated with 1.25 or 2.5 mg/kg DOX were treated on days 4, 11, and 20 post xenografts while mice treated with 5 mg/kg DOX were treated on days 4 and 11 only (and were sacrificed on day 19). Tumor volume and body weight were measured at the indicated time points (Figure 1A and Figure S1).

All untreated mice were sacrificed on day 19 when tumor diameter was >1.5 cm. In mice treated with 5, 2.5, or 1.25 mg/kg DOX, tumors grew slowly and were significantly smaller than tumors in untreated mice (*p* = 0.0081 (n = 3), 0.0087 (n = 4), and 0.0044 (n = 4), respectively). Changes in body weight are an essential parameter for evaluating systemic toxicity. All mice treated with 5 mg DOX were sacrificed by day 19 (without the 3rd DOX injection) due to body weight loss (>20%) (Figure 1B). The mice also exhibited weakened movement and vitality. Figure 1B shows that the body weight of mice treated with 1.25 mg/kg DOX increased throughout the experiment. The mice also remained vigorous and had a healthy appearance. In contrast, a notable body weight loss was observed in the 2.5 mg/kg DOX-treated group (*p* = 0.047, n = 4) (Figure 1B). Moreover, the mice in this group exhibited weakened movement and vitality.

The 5 mg/kg dose was specifically chosen to define the in vivo toxicity threshold, consistent with published data [25,26]. As expected, toxicity was high at 5 mg/kg and intermediate at 2.5 mg/kg, with significant weight loss observed at the highest dose. All DOX-treated groups showed delayed tumor growth, but 1.25 mg/kg provided optimal efficacy with minimal toxicity, aligning with prior reports [27]. Based on these findings, we used 1.25 mg/kg in subsequent experiments. Our experimental design included a high-dose arm to outline toxicity limits and lower-dose arms (1.25–2.5 mg/kg) to assess efficacy with tolerability.

### 3.2. PLGA–OCA May Augment Immune Responses and Contribute to Tumor-Cell Death

To improve the delivery and efficacy of DOX for tumor treatment, we conjugated DOX onto PLGA surface-activated nanoparticles. We previously employed PLGA-NPs in vivo to evaluate their safety and found that neither bare PLGA-NPs [28] nor PLGA-OCA (PLGA-NPs with the OCA linker) [20] caused any detectable toxicity in mice, as evidenced by normal hematological, clinical chemistry, and histopathological assessments [20,28].

**Figure 2:**
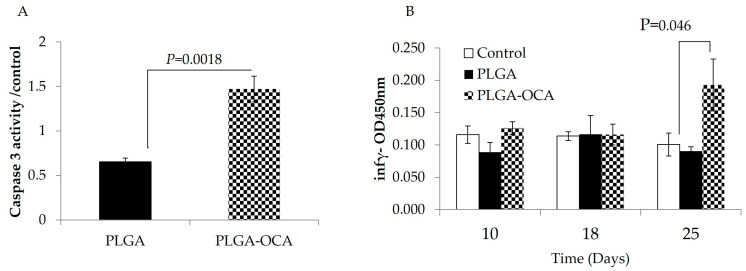
OCI-Ly19 DLBCL cells were injected subcutaneously into the right flank of NOD/SCID mice. Mice were treated with PLGA-NPs (n = 10) or PLGA-OCA (n = 10) on days 4, 11, and 20 after tumor cell injection. (**A**) Caspase-3 activity in crude protein lysates collected from subcutaneous tumors. (**B**) Serum IFN-γ levels were determined by drawing blood at the indicated days; 5 μL of serum were assayed for IFN-γ.

To test the effect of the OCA linker in our model, xenograft mice were treated once a week with either PLGA-NPs or PLGA-OCA on days 4, 11, and 20 after tumor cell injection. On day 25, all treated mice were sacrificed. Subcutaneous tumors were removed, and crude protein from tumor lysates was analyzed for caspase-3 activity, an indicator of apoptotic cell death. Caspase-3 activity in tumors from mice treated with PLGA-OCA was 2.5-fold greater compared to mice treated with PLGA-NPs (*p* = 0.0018) (Figure 2A). This indicates that PLGA-OCA induces robust apoptotic cell death and tumor destruction, confirming the ability of these functionalized nanoparticles to kill tumor cells.

**Figure 3:**
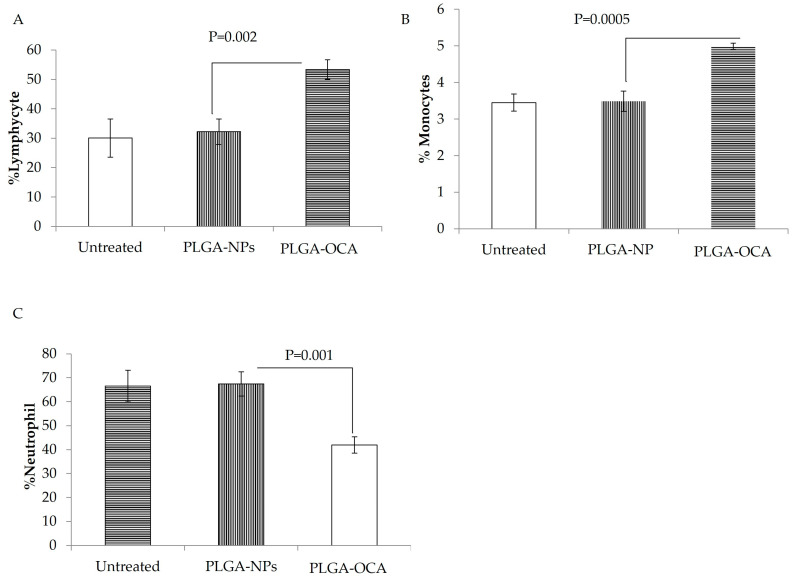
Mice were treated once a week with PLGA-NPs or PLGA-OCA-NPs (total of 3 treatments). One day after the third treatment, mice were bled from the facial vein, and the percentage of (**A**) neutrophils, (**B**) lymphocytes, and (**C**) monocytes was determined in the peripheral blood.

PLGA-OCA treatment appeared to trigger an immune response even in immunodeficient NOD/SCID mice, as reflected by elevated interferon-γ levels in the serum. This increase in IFN-γ is likely driven by natural killer (NK) cells, since these mice lack functional T and B lymphocytes and NK cells are the primary remaining immune effectors [29]. At the end of the experiment (25 days), significantly high levels of IFN-γ were detected in the serum of mice treated with PLGA-OCA (*p* = 0.046 compared to PLGA-NPs–treated), while in PLGA-NPs–treated mice, IFN-γ serum levels were the same as those of untreated mice (Figure 2B). Complete blood count (CBC) shows a percentage increase in lymphocytes and monocytes in mice treated with PLGA-OCA compared to PLGA-NPs–treated mice and a decrease in neutrophil cells (Figure 3). This result shows that INF-γ levels increased in PLGA-OCA–treated mice in association with immune system cell response.

### 3.3. DOX-OCA Induces Cell Death In Vitro and In Vivo

**Figure 4:**
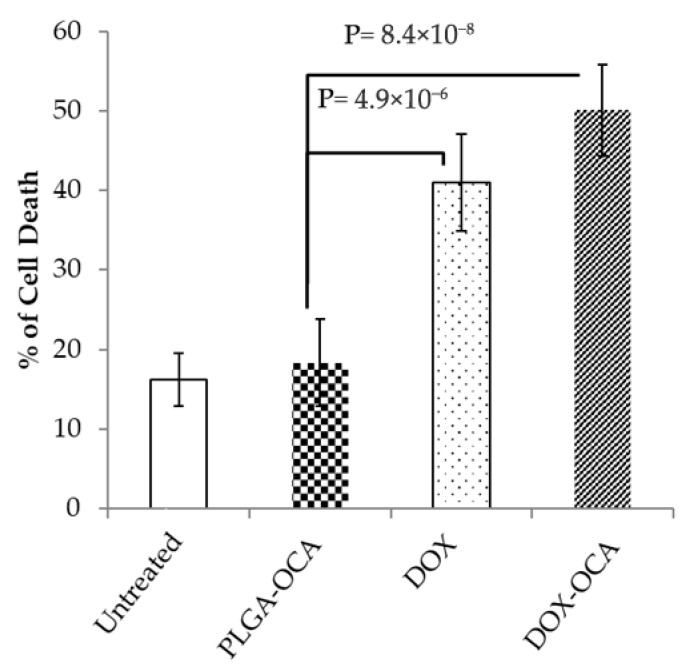
OCI-Ly19 DLBCL cells were treated for 48 h with PLGA–OCA (5 µg), DOX–OCA (2 µM DOX encapsulated in 5 µg PLGA), or free DOX (2 µM). Cell death was assessed by flow cytometry using annexin V and propidium iodide (PI) staining, comprising annexin V^+^ and annexin V^+^/PI+ cells (n = 5 replicates).

To improve the delivery and efficacy of DOX for the treatment of tumors, we next conjugated DOX onto PLGA-OCA NPs (DOX-OCA) (see Figure S2 and Supplementary Table S1), and OCI-Ly19 DLBCL cells were used to evaluate the effect of DOX-OCA on cell survival. Treatment with DOX (2 µM, 24 h) significantly induced cell death in OCI-Ly19 as determined by annexin V staining and flow cytometry (*p* = 4.9 × 10^−6^, n = 5 replicates). DOX-OCA (2 µM DOX encapsulated in 5 µg PLGA, 48 h) also induced highly significant cell death of OCI-Ly19 (*p* = 8.4 × 10^−8^, n = 5 replicates) (Figure 4, Figure S3 shows a representative dot plot illustrating the cell death gating strategy).

**Figure 5:**
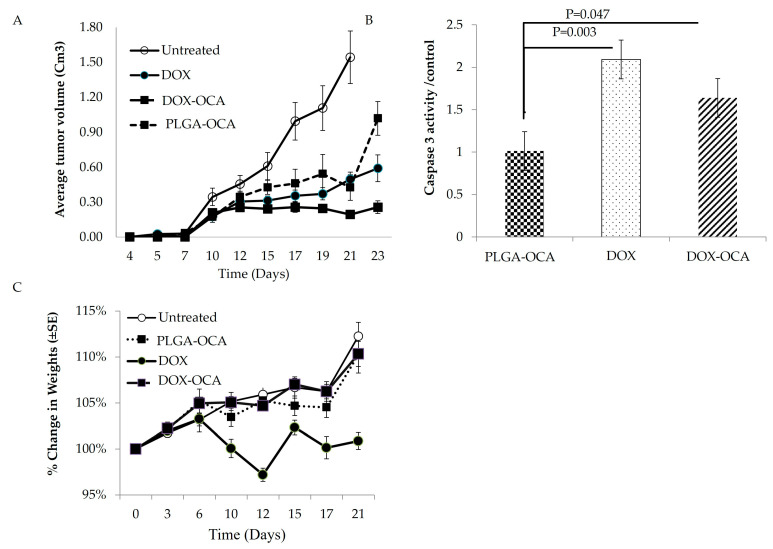
OCI-Ly19 DLBLC cells were injected subcutaneously into the right flank of NOD/SCID mice. Mice were treated (n = 6 for each group of treatment) with PLGA-OCA, DOX-OCA, or free DOX (1.25 mg/kg) on days 4, 11, and 20 after tumor cell injection or left untreated. (**A**) Tumor measurements were obtained every 2 to 3 days. (**B**) At sacrifice, tumors were lysed, and caspase-3 activity was quantified in the lysates. (**C**) Change in body weight during the experiment (21 days).

The therapeutic potential of DOX-OCA treatment was evaluated in vivo using the DLBCL model [24]. Mice were injected subcutaneously with OCI-Ly19 cells, and tumor volume was measured (n = 10). On days 4, 11, and 20, mice were treated with PLGA-OCA (0.5 mg PLGA-OCA), DOX (1.25 mg/kg), or DOX-OCA (1.25 mg/kg DOX/0.5 mg PLGA-OCA) or left untreated (Figure 5).

Untreated controls died due to tumor diameter exceeding 1.5 cm by 19–21 days after tumor cell subcutaneous injection; treated mice were sacrificed at days 21–23.

Significant antitumor activity was observed in mice treated with DOX-OCA, as evidenced by smaller tumor volumes compared to the PLGA-OCA-only group on days 21 and 23 (*p* = 0.00196, n = 6) ( Figure 5A and Figure S4). DOX-OCA–treated tumors grew more slowly compared to controls, and by days 21–23 post-injection, they were significantly smaller than tumors in mice treated with free DOX (*p* = 0.034, n = 6). Subcutaneous tumors were excised at sacrifice, and tumor lysates were analyzed for caspase-3 activity as a readout of apoptotic cell death. High caspase-3 activity was detected in tumors from mice treated with free DOX and DOX-OCA (*p* = 0.030 and 0.047 vs. PLGA-OCA, respectively; n = 6) (Figure 5B), indicating that DOX-OCA treatment effectively induces cell death in tumor cells. Importantly, these results confirm that the DOX-loaded nanoparticles successfully targeted the tumors [24] (Figure 5).

Change in body weight is an important parameter to evaluate systemic toxicity of treatment. Throughout the experiment, the free DOX-treated group showed a notable loss in body weight (Figure 5C; *p* = 2.7 × 10^–3^, n = 6) compared to free Dox treatment. Mice in this group also exhibited reduced movement and vitality, consistent with higher systemic toxicity. In contrast, the DOX-OCA–treated mice maintained body weight and appeared more active, suggesting improved tolerability.

## 4. Discussion

Doxorubicin (DOX) has anchored DLBCL therapy for almost five decades, yet dose-dependent cardiomyopathy and the evolutionary rise in drug-resistant clones keep cure rates well below their theoretical ceiling [1,4,8,9]. First-generation nanocarriers such as pegylated liposomal DOX (PL-DOX; Doxil^®^) were introduced to spare the myocardium; meta-analyses document a three- to four-fold drop in grade ≥ 3 cardiotoxic events compared with conventional DOX [12,30]. Clinical benefit in aggressive lymphomas, however, has remained modest: objective responses in relapsed or refractory (R/R) DLBCL rarely exceed 30%, and cardiotoxicity, although attenuated, still accumulates with successive cycles [1,31]. Two shortcomings dominate the mechanistic discussion—premature drug leakage from liposomes in inflammatory plasma and poor penetration beyond perivascular niches in densely cellular lymphomas [14,15].

Our group previously used a PLGA scaffold to deliver CD40-targeted mini-tLivin nanoparticles, achieving durable tumor regression in a disseminated DLBCL mouse model [24]. Building on that success, we set out to widen the therapeutic window of DOX with the same biodegradable platform. PLGA nanoparticles already outperform liposomes in several solid-tumor models by prolonging DOX half-life four- to six-fold and lowering systemic exposure [32,33], yet lymphoma-specific data remain scarce. Our group provides the only ligand-directed PLGA report in disseminated DLBCL to date [24]. We therefore engineered DOX-loaded PLGA particles coated with an Oleyl cysteineamide shell (Figure 2), hereafter termed PLGA–OCA. Beyond colloidal stabilization, the OCA layer confers mild immunoadjuvant properties (Figure 2) absent from liposomes and conventional PLGA carriers [28,34].

Head-to-head dose-finding in OCI-Ly19 xenografts, a TP53-wild, double-hit, germinal-center model that typifies high-risk clinical disease [21,22,23], reproduced the classic biphasic toxicity of free DOX (Figure 1): doses ≥ 2.5 mg kg^−1^ produced weight loss, whereas 1.25 mg kg^−1^ delivered maximal tumor control with tolerable weight loss, echoing historical observations in other GCB lymphoma [35]. Encapsulation of the same payload in PLGA–OCA pushed efficacy and safety further (Figure 5): tumor-doubling time lengthened by 55%, and treatment-related weight loss was abolished, outperforming both liposomal DOX (18% delay in comparable xenografts [28]) and antibody-decorated PLGA constructs (40% delay [20]).

Mechanistic readouts support a dual mode of action; intertumoral cleaved-caspase-3 doubled relative to free DOX and more than compensated for the reduction in systemic exposure, in line with CREB3L1-mediated chemosensitivity [5,6,8,32]. In parallel, PLGA–OCA produced a 1.8-fold rise in INF-γ levels, an effect not reported for liposomal or unmodified PLGA vehicles (Figure 2). Although the innate signal recorded here was modest, likely dominated by natural-killer activity in NOD/SCID hosts [29], it did not compromise the compatibility of PLGA–OCA with monoclonal antibodies, CAR-T products, or PD-1 antagonists that are now standard after R-CHOP failure [4,5,31,36,37].

Several translational hurdles remain. Comprehensive biodistribution, including quantitative cardiac and marrow uptake, is essential to confirm the anatomical basis of cardioprotection. Activated-B-cell DLBCL, whose vasculature yields a less pronounced enhanced-permeability-and-retention benefit, must be evaluated. A phase Ib dose-escalation trial in R/R DLBCL, stratified by TP53 status, CREB3L1 expression, and the revised International Prognostic Index [35,38], would clarify feasibility and enable combination with PD-1 blockade to determine whether the observed innate activation augments adaptive immunity.

Combining a biodegradable PLGA core with an OCA shell presents a novel approach to addressing two significant challenges in doxorubicin therapy: the short circulation of the drug and a weak immune response. Our results show that DOX-loaded PLGA–OCA nanoparticles improve tumor control and reduce toxicity compared to free DOX, expanding the therapeutic window beyond that of liposomes or antibody-decorated PLGA. However, these results are preliminary and based on a single model. Further studies in different models (p53-deficient or mutant DLBCL models), with more extended follow-up periods, comprehensive pharmacokinetic analysis, and large-scale production, are needed to confirm these findings and support clinical development.

## 5. Conclusions

PLGA-OCA nanoparticles containing 1.25 mg/kg of doxorubicin significantly reduce tumor growth in OCI-Ly19 compared to the free drug. DOX-OCA also eliminates the toxicity and weight loss typically associated with conventional dosing. We observed increased caspase-3 activity within the tumors, and PLGA-OCA influenced the elevation of IFN-γ, indicating both cytotoxic effects and activation of the innate immune response. These findings provide a rationale for further evaluating this nanoparticle platform in genetically diverse DLBCL subtypes, including those less responsive to conventional therapies.

## Data Availability

The original contributions presented in this study are included in the article. Further inquiries can be directed to the corresponding author.

## Supplementary Materials

The following supporting information can be downloaded at: https://www.mdpi.com/article/10.3390/cells14171334/s1, Figure S1: Representative images of excised tumors from control mice and those treated with different doses of DOX; Figure S2: Calibration curve of doxorubicin as determined by UV absorbance at 475 nm; Figure S3: Measuring cell death by annexin V/propidium iodide (PI) staining following treatment with nanoparticles and/or chemotherapy Figure S4: Representative images of excised OCI-LY19 tumors following in vivo treatment; Table S1: Physicochemical properties of the various protein-loaded PLGA nanoparticles formulations. The encapsulation yield is low (45%) due to the hydrophilic nature of the doxorubicin, and the physicochemical properties as depicted in Table S1, show no change in zeta potential in the various NPs formulations.

## References

[1] Foo J., Michor F. (2014). Evolution of acquired resistance to anti-cancer therapy. J. Theor. Biol..

[2] Girotti M.R., Marais R. (2013). Deja Vu: EGF receptors drive resistance to BRAF inhibitors. Cancer Discov..

[3] Susanibar-Adaniya S., Barta S.K. (2021). 2021 Update on Diffuse large B cell lymphoma: A review of current data and potential applications on risk stratification and management. Am. J. Hematol..

[4] Coiffier B., Sarkozy C. (2016). Diffuse large B-cell lymphoma: R-CHOP failure-what to do?. Hematol. Am. Soc. Hematol. Educ. Program..

[5] Chavez J.C., Bachmeier C., Kharfan-Dabaja M.A. (2019). CAR T-cell therapy for B-cell lymphomas: Clinical trial results of available products. Ther. Adv. Hematol..

[6] Chatterjee K., Zhang J., Honbo N., Karliner J.S. (2010). Doxorubicin cardiomyopathy. Cardiology.

[7] Denard B., Lee C., Ye J. (2012). Doxorubicin blocks proliferation of cancer cells through proteolytic activation of CREB3L1. eLife.

[8] Denard B., Pavia-Jimenez A., Chen W., Williams N.S., Naina H., Collins R., Brugarolas J., Ye J. (2015). Identification of CREB3L1 as a Biomarker Predicting Doxorubicin Treatment Outcome. PLoS ONE.

[9] Lüpertz R., Wätjen W., Kahl R., Chovolou Y. (2010). Dose- and time-dependent effects of doxorubicin on cytotoxicity, cell cycle and apoptotic cell death in human colon cancer cells. Toxicology.

[10] Singal P.K., Iliskovic N. (1998). Doxorubicin-induced cardiomyopathy. N. Engl. J. Med..

[11] Carvalho C., Santos R.X., Cardoso S., Correia S., Oliveira P.J., Santos M.S., Moreira P.I. (2009). Doxorubicin: The good, the bad and the ugly effect. Curr. Med. Chem..

[12] Rafiyath S.M., Rasul M., Lee B., Wei G., Lamba G., Liu D. (2012). Comparison of safety and toxicity of liposomal doxorubicin vs. conventional anthracyclines: A meta-analysis. Exp. Hematol. Oncol..

[13] Lu Y., Chen S.C. (2004). Micro and nano-fabrication of biodegradable polymers for drug delivery. Adv. Drug Deliv. Rev..

[14] Fang J., Nakamura H., Maeda H. (2011). The EPR effect: Unique features of tumor blood vessels for drug delivery, factors involved, and limitations and augmentation of the effect. Adv. Drug Deliv. Rev..

[15] Maeda H. (2001). The enhanced permeability and retention (EPR) effect in tumor vasculature: The key role of tumor-selective macromolecular drug targeting. Adv. Enzym. Regul..

[16] Alizadeh D., Trad M., Hanke N.T., Larmonier C.B., Janikashvili N., Bonnotte B., Katsanis E., Larmonier N. (2014). Doxorubicin eliminates myeloid-derived suppressor cells and enhances the efficacy of adoptive T-cell transfer in breast cancer. Cancer Res..

[17] Mukherjee O., Paul S., Das S., Rakshit S., Shanmugam G., George M., Sarkar K. (2024). Doxorubicin induced epigenetic regulation of dendritic cell maturation in association with T cell activation facilitates tumor protective immune response in non-small cell lung cancer (NSCLC). Pathol. Res. Pr..

[18] Jia J., Xiong Z.A., Qin Q., Yao C.G., Zhao X.Z. (2015). Picosecond pulsed electric fields induce apoptosis in a cervical cancer xenograft. Mol. Med. Rep..

[19] Fessi H., Puisieux F., Devissaguet J.P., Ammoury N., Benita S. (1989). Nanocapsule formation by interfacial polymer deposition following solvent displacement. Int. J. Pharm..

[20] Karra N., Nassar T., Ripin A.N., Schwob O., Borlak J., Benita S. (2013). Antibody conjugated PLGA nanoparticles for targeted delivery of paclitaxel palmitate: Efficacy and biofate in a lung cancer mouse model. Small.

[21] Kubacz M., Kusowska A., Winiarska M., Bobrowicz M. (2022). In Vitro Diffuse Large B-Cell Lymphoma Cell Line Models as Tools to Investigate Novel Immunotherapeutic Strategies. Cancers.

[22] Lu T.X., Wu S., Cai D.Y., Hong T.T., Zhang Y., Gao H.Q., Hua H.Y., Wu X.H. (2019). Prognostic significance of serum aspartic transaminase in diffuse large B-cell lymphoma. BMC Cancer.

[23] Matthews J.M., Bhatt S., Patricelli M.P., Nomanbhoy T.K., Jiang X., Natkunam Y., Gentles A.J., Martinez E., Zhu D., Chapman J.R. (2016). Pathophysiological significance and therapeutic targeting of germinal center kinase in diffuse large B-cell lymphoma. Blood.

[24] Abd-Elrahman I., Nassar T., Khairi N., Perlman R., Benita S., Ben Yehuda D. (2021). Novel targeted mtLivin nanoparticles treatment for disseminated diffuse large B-cell lymphoma. Oncogene.

[25] Aston W.J., Hope D.E., Nowak A.K., Robinson B.W., Lake R.A., Lesterhuis W.J. (2017). A systematic investigation of the maximum tolerated dose of cytotoxic chemotherapy with and without supportive care in mice. BMC Cancer.

[26] Asnani A., Moslehi J.J., Adhikari B.B., Baik A.H., Beyer A.M., de Boer R.A., Ghigo A., Grumbach I.M., Jain S., Zhu H. (2021). Preclinical Models of Cancer Therapy-Associated Cardiovascular Toxicity: A Scientific Statement From the American Heart Association. Circ. Res..

[27] Wunderlich M., Manning N., Sexton C., Sabulski A., Byerly L., O’Brien E., Perentesis J.P., Mizukawa B., Mulloy J.C. (2019). Improved chemotherapy modeling with RAG-based immune deficient mice. PLoS ONE.

[28] Harush-Frenkel O., Bivas-Benita M., Nassar T., Springer C., Sherman Y., Avital A., Altschuler Y., Borlak J., Benita S. (2010). A safety and tolerability study of differently-charged nanoparticles for local pulmonary drug delivery. Toxicol. Appl. Pharmacol..

[29] Carreno B.M., Garbow J.R., Kolar G.R., Jackson E.N., Engelbach J.A., Becker-Hapak M., Carayannopoulos L.N., Piwnica-Worms D., Linette G.P. (2009). Immunodeficient mouse strains display marked variability in growth of human melanoma lung metastases. Clin. Cancer Res..

[30] Dorostkar H., Haghiralsadat B.F., Hemati M., Safari F., Hassanpour A., Naghib S.M., Roozbahani M.H., Mozafari M.R., Moradi A. (2023). Reduction of Doxorubicin-Induced Cardiotoxicity by Co-Administration of Smart Liposomal Doxorubicin and Free Quercetin: In Vitro and In Vivo Studies. Pharmaceutics.

[31] Schipani M., Rivolta G.M., Margiotta-Casaluci G., Mahmoud A.M., Al Essa W., Gaidano G., Bruna R. (2023). New Frontiers in Monoclonal Antibodies for Relapsed/Refractory Diffuse Large B-Cell Lymphoma. Cancers.

[32] Sun L., Liu H., Ye Y., Lei Y., Islam R., Tan S., Tong R., Miao Y.B., Cai L. (2023). Smart nanoparticles for cancer therapy. Signal Transduct. Target. Ther..

[33] Aryal S., Park S., Park H., Park C., Kim W.C., Thakur D., Won Y.J., Key J. (2023). Clinical Trials for Oral, Inhaled and Intravenous Drug Delivery System for Lung Cancer and Emerging Nanomedicine-Based Approaches. Int. J. Nanomed..

[34] Chehelgerdi M., Chehelgerdi M., Allela O.Q.B., Pecho R.D.C., Jayasankar N., Rao D.P., Thamaraikani T., Vasanthan M., Viktor P., Lakshmaiya N. (2023). Progressing nanotechnology to improve targeted cancer treatment: Overcoming hurdles in its clinical implementation. Mol. Cancer.

[35] Lenz G., Wright G.W., Emre N.C., Kohlhammer H., Dave S.S., Davis R.E., Carty S., Lam L.T., Shaffer A.L., Xiao W. (2008). Molecular subtypes of diffuse large B-cell lymphoma arise by distinct genetic pathways. Proc. Natl. Acad. Sci. USA.

[36] Neelapu S.S., Locke F.L., Bartlett N.L., Lekakis L.J., Miklos D.B., Jacobson C.A., Braunschweig I., Oluwole O.O., Siddiqi T., Lin Y. (2017). Axicabtagene Ciloleucel CAR T-Cell Therapy in Refractory Large B-Cell Lymphoma. N. Engl. J. Med..

[37] Schuster S.J., Svoboda J., Chong E.A., Nasta S.D., Mato A.R., Anak Ö., Brogdon J.L., Pruteanu-Malinici I., Bhoj V., Landsburg D. (2017). Chimeric Antigen Receptor T Cells in Refractory B-Cell Lymphomas. N. Engl. J. Med..

[38] Sehn L.H., Berry B., Chhanabhai M., Fitzgerald C., Gill K., Hoskins P., Klasa R., Savage K.J., Shenkier T., Sutherland J. (2007). The revised International Prognostic Index (R-IPI) is a better predictor of outcome than the standard IPI for patients with diffuse large B-cell lymphoma treated with R-CHOP. Blood.

