# Supplementary material for: Doxorubicin-Loaded Nanoparticle Treatment Enhances Diffuse Large B-Cell Lymphoma Cell Death
Source: Cells. 2025 Aug 28;14(17):1334. doi: 10.3390/cells14171334 (PMC12428780; doi:10.3390/cells14171334)
Supplement: Supplementary file 1 [file cells-14-01334-s001.zip › cells-3692841-supplementary.pdf]

Supplementary figures and table:

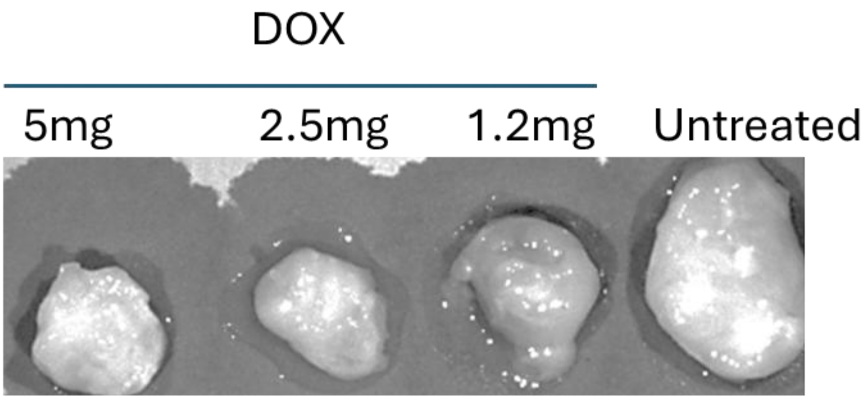

Figure S1. Representative images of excised tumors from control mice and those treated with different doses of DOX. Mice were treated with different doses of DOX, receiving either two injections of 5 mg or three injections of 2.5 mg and 1.2 mg. All untreated mice and those treated with 5 mg had to be sacrificed on day 19 following cell injection. Therfore, mice treated with 1.2 mg and 2.5 mg DOX were also sacrificed on day 19 to allow for a direct comparison of tumor sizes across the various experimental groups.

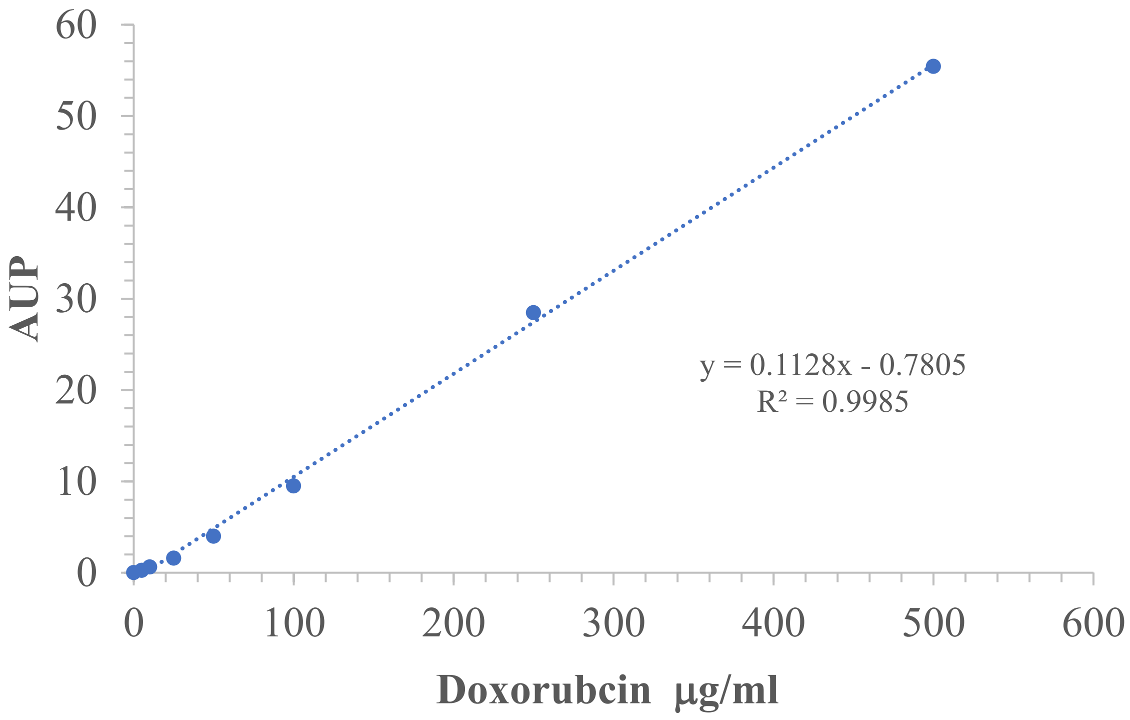

Figure S2. Calibration curve of doxorubicin as determined by UV absorbance at 475 nm. A standard curve was generated by measuring the absorbance of doxorubicin solutions ranging from 5 to 500 µg/mL in water:acetonitrile (1:1). The calibration curve ($y = 0.1128x - 0.7805$, $R^2 = 0.9985$) was used to quantify doxorubicin content in nanoparticle formulations following purification. The encapsulation yield of doxorubicin in PLGA nanoparticles was calculated as 45%, with a final doxorubicin-loaded NP concentration of 0.9 mg/mL.

Formulation	PLGA (mg/ml)	Mean diameter (nm)	Zeta potential (mv)	Doxorubicin [DOX] mg/ml
Blank-NPS	0.4	83	-31	
DOX-OCA	0.4	105	-33	0.125

Supplementary Table S1: Physicochemical properties of the various protein-loaded PLGA nanoparticles formulations. The encapsulation yield is low (45%) due to the hydrophilic nature of the doxorubicin, and the physicochemical properties as depicted in Table 1, show no change in zeta potential in the various NPs formulations.

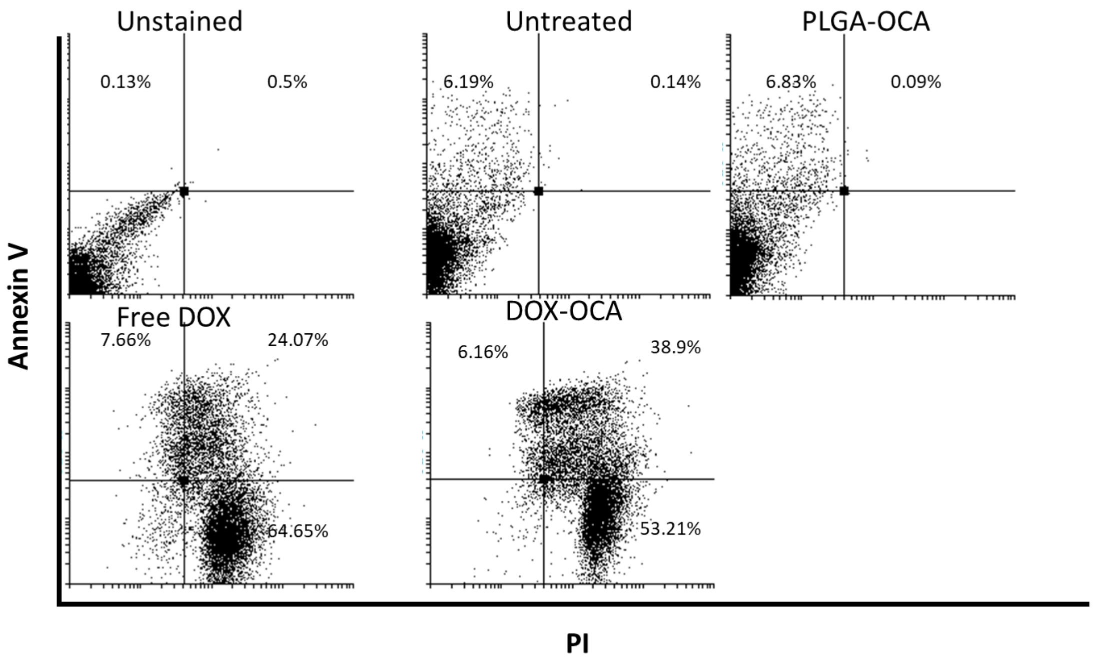

Figure S3. Measuring cell death by annexin V/propidium iodide (PI) staining following treatment with nanoparticles and/or chemotherapy.
OCI-Ly19 cells (5×10^5) were treated for 48 h, then stained with annexin V–CY5 and PI. Dot plots represent viable (Annexin V^-/PI^-; lower left), PI-positive (Annexin V^-/PI^+; lower right), late apoptotic (Annexin V^+/PI^+; upper right), and annexin V-positive cells (Annexin V^+/PI^-; upper left). Percentages in each quadrant are indicated. Treatments increased apoptotic and cell death populations compared to untreated controls.

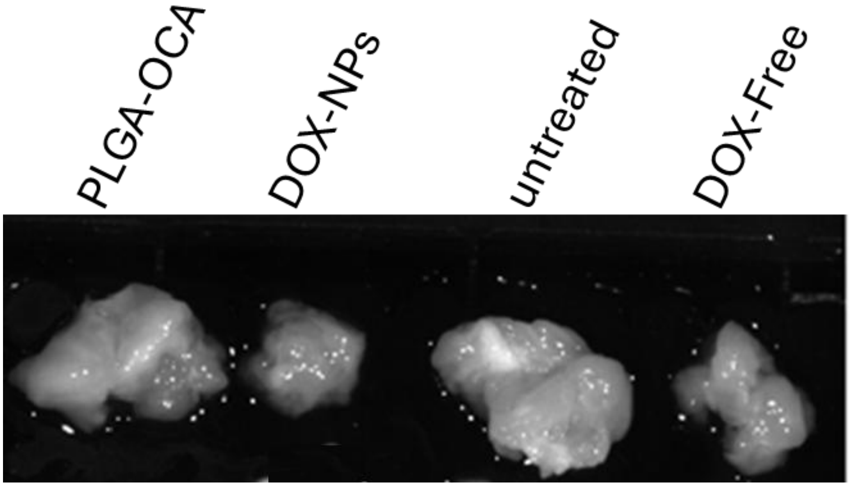

Figure S4. Representative images of excised OCI-LY19 tumors following in vivo treatment.

Tumors were removed from treated mice at day 23 post-cell injection. The untreated tumor is from a mouse that survived until 23 days post cell injection.
